# Evaluation of the global association between cholesterol-associated polymorphisms and Alzheimer's disease suggests a role for rs3846662 and *HMGCR *splicing in disease risk

**DOI:** 10.1186/1750-1326-6-62

**Published:** 2011-08-25

**Authors:** Christopher R Simmons, Fanggeng Zou, Steven G Younkin, Steven Estus

**Affiliations:** 1 Department of Physiology, Sanders-Brown Center on Aging, University of Kentucky, Lexington, KY, USA; 2 Department of Neuroscience, Mayo Clinic College of Medicine, Jacksonville, FL, USA

**Keywords:** Alzheimer, genetics, cholesterol, GWAS, SNP, HMGCR, statin

## Abstract

**Background:**

Recent genome-wide association studies (GWAS) have identified single nucleotide polymorphisms (SNP)s that are essentially unequivocally associated with peripheral cholesterol. Since the alleles of the *APOE *gene, which modulate peripheral cholesterol metabolism, and midlife plasma cholesterol are both associated with Alzheimer's disease (AD) risk, we have evaluated the hypothesis that SNPs associated with plasma cholesterol are also associated with AD.

**Results:**

Seventeen non-*APOE *SNPs reproducibly associated with cholesterol per GWAS were tested for association with AD in ~2,000 AD and ~4,000 non-AD subjects. As a group, these SNPs are associated with AD. Two SNPs in particular, rs3846662 and rs1532085, are associated with AD risk and age-of-onset. Additionally, rs3846662 was associated with *HMGCR *exon 13 splicing in human liver but not brain, possibly obscured by CNS cell-type heterogeneity. However, rs3846662 was associated with *HMGCR *exon 13 splicing in liver- and brain-derived cell lines.

**Conclusions:**

Cholesterol-associated SNPs outside of *APOE *confer a global risk for AD. Rs3846662 and rs1532085 are associated with both AD risk and age-of-onset. Rs3846662 is associated with *HMGCR *exon 13 inclusion. Since rs3846662 affects AD risk and age-of-onset as well as statin responsiveness, this SNP may confound clinical trials evaluating the protective effects of statins on AD.

## Background

Late-onset Alzheimer's disease (AD) is a devastating form of dementia with no clear etiology. As much as 80% of age-adjusted AD risk may be genetic based upon studies of monozygotic twins [1,2]. The primary genetic modulators of AD risk are the alleles of the gene encoding apolipoprotein E (*APOE*), i.e. *APOE*-ε2, *APOE*-ε3 and *APOE*-ε4. The presence of *APOE*-ε4 alone accounts for up to 53% of AD risk while the presence of *APOE*-ε2 is protective against the disease [3-5].

Multiple theories have been proposed to account for the mechanism underlying the association of APOE with AD [6,7]. One theory suggests that the alleles of *APOE *modulate AD risk via their effects on cholesterol homeostasis. This is supported by evidence that the *APOE*-ε4 and *APOE*-ε2 alleles are associated with increased and decreased levels of plasma cholesterol, respectively [8]. Furthermore, elevated midlife cholesterol is itself a risk factor for AD, raising the possibility that genetic modulators of cholesterol may also modulate AD risk [9,10]. To this end, prior studies have demonstrated variable associations between polymorphisms in genes related to cholesterol metabolism and AD risk [11-13]. However, ontological analysis indicates that genes involved in cholesterol metabolism are significantly overrepresented as being associated with AD [14]. Genome-wide association studies (GWAS)s have recently uncovered single nucleotide polymorphisms (SNP)s that are robustly and reproducibly associated with total cholesterol, low-density lipoprotein (LDL) and/or high-density lipoprotein (HDL) [15-23]. As such, these SNPs are essentially unequivocally associated with cholesterol and hence serve as outstanding tools to evaluate the genetic relatedness between cholesterol and AD. Thus, based on (i) the significant heritability of AD, (ii) the robust involvement of *APOE *in AD risk, (iii) a growing body of evidence that cholesterol itself modulates AD risk and (iv) the ontological overrepresentation of cholesterol gene variants in AD GWAS results we hypothesize that SNPs associated with peripheral cholesterol via GWAS also contribute to AD risk.

To test this hypothesis, we evaluated SNPs associated with plasma total cholesterol, LDL and HDL for their association with AD risk. Our results indicate that, as a group, these SNPs are significantly associated with AD. Additionally, rs3846662 and rs1532085 are also associated with AD age-of-onset. *In vivo*, rs3846662 is associated with *HMGCR *exon 13 inclusion in human liver but lacks a clear association with *HMGCR *splicing in human brain. HMGCR staining in brain indicated that the enzyme is expressed in both neurons and glia. Thus, we evaluated the function of rs3846662 *in vitro *using human liver- and CNS-derived cell lines, where rs3846662 was associated with the splicing efficiency of *HMGCR *exon 13 in both cell types. In conclusion, cholesterol-associated SNPs identified by GWAS, as a group, are associated with AD and exhibit the potential to elucidate novel mechanisms underlying AD risk and age-of onset.

## Results

### Cholesterol-associated SNPs are also associated with AD

A review of GWAS via the HuGE Navigator database identified eighteen non-redundant SNPs whose associations with total cholesterol, LDL and/or HDL are highly significant (p < 1 × 10^-10^) and have been replicated in at least two populations. To assess the contribution of these SNPs to AD risk we queried their association with AD using a three-phase approach.

Phase 1 association testing between these eighteen cholesterol-associated SNPs and AD was performed by using 843 AD and 1,264 non-AD samples. PLINK was used to test for SNP-AD associations per additive models for the eighteen cholesterol-associated SNPs. Of the eighteen SNPs, rs157580 is in linkage with *APOE *and served as a positive control for AD association. In our Phase I study population, rs157580 and was significantly associated with AD in an additive model (p = 3.0 × 10^-22^, OR = 0.51). Henceforth only seventeen cholesterol-associated SNPs were considered for the purpose of multiple testing.

Global analysis of all seventeen cholesterol-associated SNPs revealed that, as a group, these SNPs are significantly associated with AD (p = 0.017, Table 1). Furthermore, two of these SNPs exhibited nominally significant associations with AD (p ≤ 0.05, Table 1). These SNPs are located in or near the genes *HMGCR *(rs3846662) and *MMAB/MVK *(rs2338104). Three additional SNPs exhibited trends with AD (p < 0.1), including rs1363232 (*TIMD4/HAVCR1*), rs1532085 (*LIPC*) and rs9989419 (*CETP*).

**Table 1:** Phase 1 analysis of association between cholesterol SNPs and AD.

CHR	GENE	SNP	OR	L95	U95	P
**19**	*APOE*	rs157580	0.51	0.45	0.59	*3.30E-21*
**5**	*HMGCR*	rs3846662	1.16	1.02	1.32	*0.02*
**12**	*MMAB/MVK*	rs7298565	0.88	0.78	1	*0.05*
**5**	*TIMD4/HAVCR1*	rs1363232	0.89	0.78	1.01	0.06
**15**	*LIPC*	rs1532085	0.89	0.78	1.01	0.07
**16**	*CETP*	rs9989419	1.12	0.98	1.27	0.09
**19**	*NCAN*	rs2304130	1.16	0.92	1.47	0.21
**20**	*HNF4A*	rs1800961	0.8	0.55	1.16	0.24
**18**	*LIPG*	rs4939883	1.09	0.92	1.28	0.32
**16**	*LCAT*	rs2271293	0.91	0.75	1.1	0.33
**1**	*SORT1*	rs646776	1.07	0.92	1.24	0.4
**1**	*GALNT2*	rs10779835	0.95	0.84	1.08	0.44
**9**	*TTC39B*	rs471364	1.07	0.88	1.3	0.48
**2**	*APOB*	rs506585	1.05	0.9	1.23	0.53
**19**	*LDLR*	rs2228671	0.96	0.79	1.17	0.66
**2**	*APOB*	rs693	0.98	0.87	1.11	0.78
**16**	*CETP*	rs3764261	0.99	0.86	1.13	0.86
**9**	*ABCA1*	rs3847303	0.99	0.82	1.19	0.92

Overall, the seventeen SNPs not in linkage with *APOE *exhibited a global association with AD by using multivariable logistic regression (p = 0.017). Two SNPs, not including rs157580 (which marks *APOE*), were nominally associated with AD per additive logistic regression modeling (p ≤ 0.05). Three other SNPs showed a trend with AD (p < 0.1).

Three of the SNPs that demonstrated a nominal association or trend with AD (p < 0.1) have also been associated with gene regulation. Rs3846662 has been associated with statin responsiveness via an intermediate effect on *HMGCR *exon 13 alternative splicing [24,25]. Rs2338104 is associated with *MMAB *mRNA and protein levels in human liver [26]. Rs1532085 has been implicated in *LIPC *expression in human liver [27].

To focus upon SNPs with the potential to provide mechanistic insights into AD risk, we pursued these three SNPs which (i) demonstrated nominally significant association or a trend with AD in Phase 1 and (ii) have been implicated in gene regulation. Thus, in Phase 2 of our study, we evaluated rs3846662, rs2338104 and rs1532085 in 1,097 AD and 2,661 non-AD samples for their association with AD. Although Phase 2 associations with AD did not reach significance, these results showed a similar trend in odds ratios for rs3846662 and rs1532085. In contrast, the minor allele of rs7298565, which was associated with decreased AD risk in Phase 1, showed an opposite trend towards an increased AD risk in Phase 2 (Table 2).

**Table 2:** Phase 2 analysis of association between cholesterol SNPs and AD.

CHR	GENE	SNP	OR	L95	U95	P
**5**	*HMGCR*	rs3846662	1.08	0.98	1.19	0.13
**12**	*MMAB/MVK*	rs7298565	1.08	0.98	1.19	0.13
**15**	*LIPC*	rs1532085	0.95	0.86	1.05	0.30

The three SNPs from Phase 1 that were either nominally associated or trended with AD (p < 0.1) and are associated with gene regulation were tested for their association with AD in a second case-control population. Both rs3846662 and rs1523085 exhibit OR's that are consistent with Phase 1 results, whereas rs7298565 exhibits an inverse OR from that of Phase 1, i.e. the minor allele of rs7298565 is associated with decreased AD risk in Phase 1 and increased AD risk in Phase 2).

**Figure 1:**
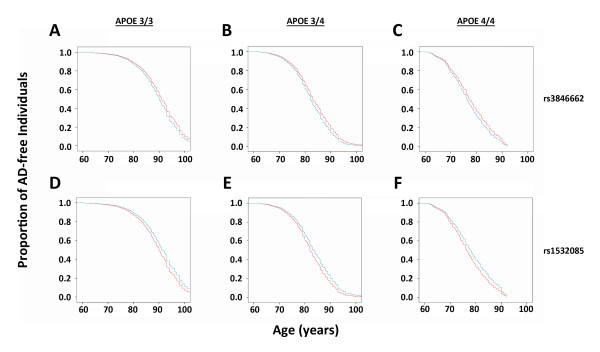
**Both rs3846662 and rs1532085 are significantly associated with AD age-of-onset**. **(A-C) **Carriers of the rs3846662_G allele exhibit a significantly earlier onset of AD than AA homozygotes, stratified by the number of *APOE-ε4 *alleles present. **(D-F) **Individuals homozygous for the A allele of rs1532085 exhibit a significantly later onset of AD than carriers of the G allele, again stratified by the number of *APOE-ε4 *alleles present. (A/D - no *APOE-ε4*, B/E - 1 copy of *APOE-ε4*, C/F - 2 copies of *APOE-ε4*)

Since our Phase 1 and Phase 2 populations were drawn from the same larger series, and to obtain maximum statistical power, we also analyzed a combined Phase 1 and Phase 2 population. Analysis of these 1,940 AD and 3,925 non-AD samples suggested that both rs3846662 and rs1532085 are associated with AD (p = 0.004 and p = 0.03, respectively, Table 3). Moreover, since the alleles of *APOE *demonstrate an effect on age of AD onset, we sought to gain further insights into the actions of rs3846662 and rs1532085 by testing for their effects on age-of-onset [5]. After stratifying for copies of the *APOE*-ε4 allele, carriers of the rs3846662_G allele have a significantly earlier onset of AD compared to non-carriers (p = 0.003, OR = 1.141, Figure 1A-C). Likewise, individuals homozygous for the rs1532085_A allele had a significantly later onset of AD than carriers of the G allele (p = 0.005, OR = 0.788, Figure 1D-F). Thus, both rs3846662 and rs1532085 are significantly associated AD age-of-onset.

**Table 3:** Phase 3 analysis of association between cholesterol SNPs and AD.

CHR	GENE	SNP	OR	L95	U95	P
**5**	*HMGCR*	rs3846662	1.12	1.04	1.21	*0.004*
**15**	*LIPC*	rs1532085	0.92	0.85	0.99	*0.03*
**12**	*MMAB/MVK*	rs7298565	0.99	0.92	1.07	0.88

Phase 1 and Phase 2 populations were combined to allow for testing of SNP-AD associations under maximal power (80% for rs3846662 and 98% for rs1532085). Both rs3846662 and rs1532085 exhibited significant associations with AD in this combined population per logistic regression (p ≤ 0.05).

### Rs3846662 and HMGCR splicing and expression in vivo

Since rs3846662 has been associated *in vitro *with *HMGCR *exon 13 splicing and with statin responsiveness, and since the contribution of statin pharmacotherapy to AD risk reduction is controversial, we chose to focus upon the actions of this SNP in further studies [24,25,28-30]. We began by testing for an association between rs3846662 and *HMGCR *exon 13 splicing *in vivo *using cDNA from human liver and brain, both of which are major cholesterol biosynthetic organs. *HMGCR *splicing was quantified as the percentage of *HMGCRΔ13 *out of total *HMGCR *message (calculated as *HMGCRΔ13 */(*HMGCRΔ13 *+ *HMGCR_FL*)).

**Figure 2:**
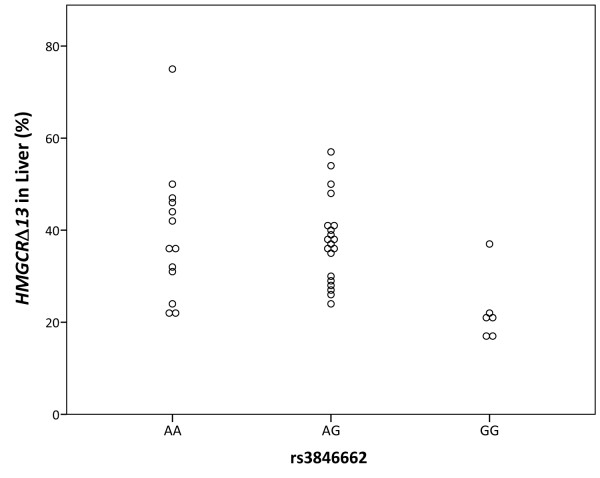
**Rs3846662 is significantly associated with percent *HMGCRΔ13 *in human liver**. Individuals homozygous for the G allele of rs3846662 exhibit the most efficient splicing of *HMGCR *exon 13 (p = 0.026, ANOVA. We note that GG homozygotes also exhibit an increased risk of AD and exhibit increased total and LDL cholesterol.

**Figure 3:**
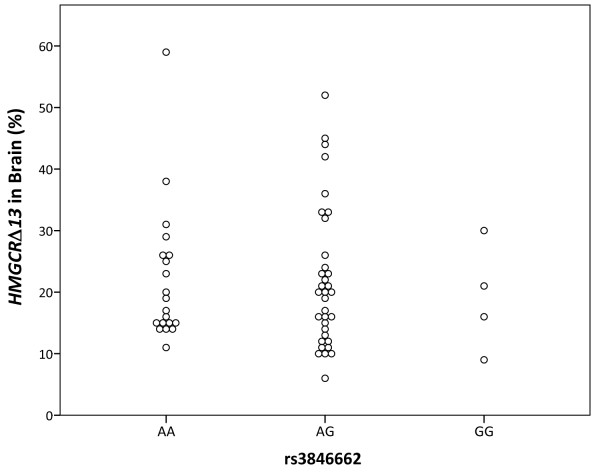
**Rs3846662 is not associated with percent *HMGCRΔ13 *in human brain**. However, we are underpowered to observe a significant difference (p ≤ 0.05) in percent *HMGCRΔ13 *between rs3846662 genotypes given the overall high level of exon 13 inclusion in brain cDNA and the possibility that rs3846662 may affect HMGCR exon 13 in a cell-type dependent manner.

In liver, we found that rs3846662 was significantly associated with *HMGCR *exon 13 splicing (p = 0.026 per ANOVA, Figure 2). Overall, *HMGCRΔ13 *represented 35.8 ± 12.2% of total *HMGCR *mRNA, with average percent *HMGCRΔ13 *values for rs3846662 AA and GG individuals differing by 16.5%. In comparison, human brain was significantly more efficient at retaining exon 13 (p < 0.001, Student's t-test), with the average percentage of *HMGCRΔ13 *being 21.8 ± 11.1%. Moreover, a trend was not detected between rs3846662 and *HMGCRΔ13 *percentage in the brain (Figure 3). In considering this result, we noted that *HMGCR *exon 13 splicing varies substantially between tissues, likely due to variation in underlying splicing factors [31]. Since brain is a particularly cell-type heterogeneous tissue, we hypothesized that our ability to detect an association between rs3846662 and *HMGCR *exon 13 splicing in human brain is confounded by *HMGCR *expression in multiple cell types within the brain.

**Figure 4:**
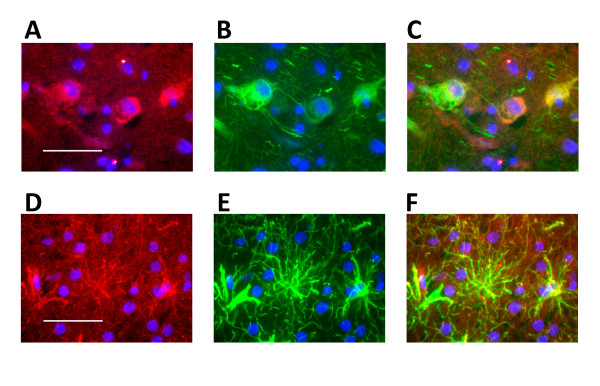
**Immunostaining reveals that HMGCR is present in both neurons and glia**. **(A-C) **HMGCR (A) is expressed within MAP2 labeled neurons (B) per merging of the two images (C). **(D-F) **Likewise, HMGCR (D) is also expressed in GFAP positive glia (E) per merging of the two images (F). Size bars in A and D represent 50 μm.

**Figure 5:**
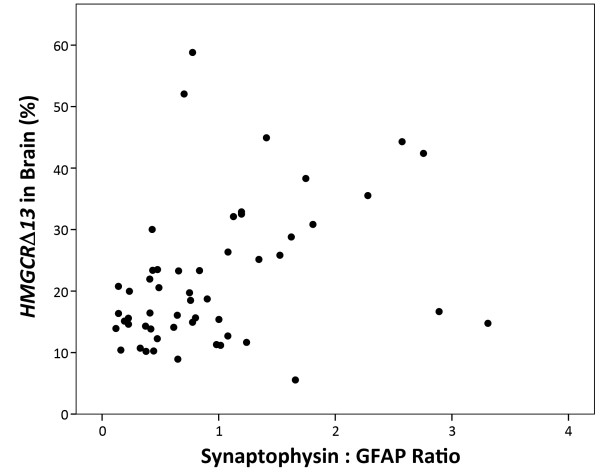
**HMGCR exon 13 splicing efficiency is associated with the ratio of neuronal to glial markers**. The percentage of *HMGCRΔ13 *in brain is significantly correlated with neuronal versus astrocyte enrichment in brain samples as determined by the ratio of synaptophysin: GFAP expression (p = 0.026, Pearson correlation = 0.339).

To investigate this hypothesis, we performed HMGCR immunostaining in human anterior cingulate. Both neurons and astrocytes expressed HMGCR as indicated by HMGCR co-localization with a neuron-specific protein, e.g., MAP2 (Figure 4A) and an astrocyte-specific protein, e.g., GFAP (Figure 4B). To evaluate whether *HMGCR *splicing may vary as a function of cell-type, we compared *HMGCR *splicing efficiency relative to the ratio of a neuron-specific mRNA (synaptophysin) and an astrocyte-specific mRNA (GFAP). This analysis revealed that as the synaptophysin: GFAP ratio increased, the percentage of *HMGCRΔ13 *increased (Figure 5); linear regression analysis found that the percentage of *HMGCRΔ13 *was significantly associated with the synaptophysin: GFAP ratio (p = 0.026) while rs3846662 and AD were not significant (p > 0.05). We speculate that variation in exon 13 splicing as a function of additional cell types and cell-specific patterns of splicing factor expression may yet obscure our ability to detect an association between rs3846662 and *HMGCR *exon 13 splicing in human brain.

### Effect of rs3846662 on splicing in-vitro using liver and brain cell lines

**Figure 6:**
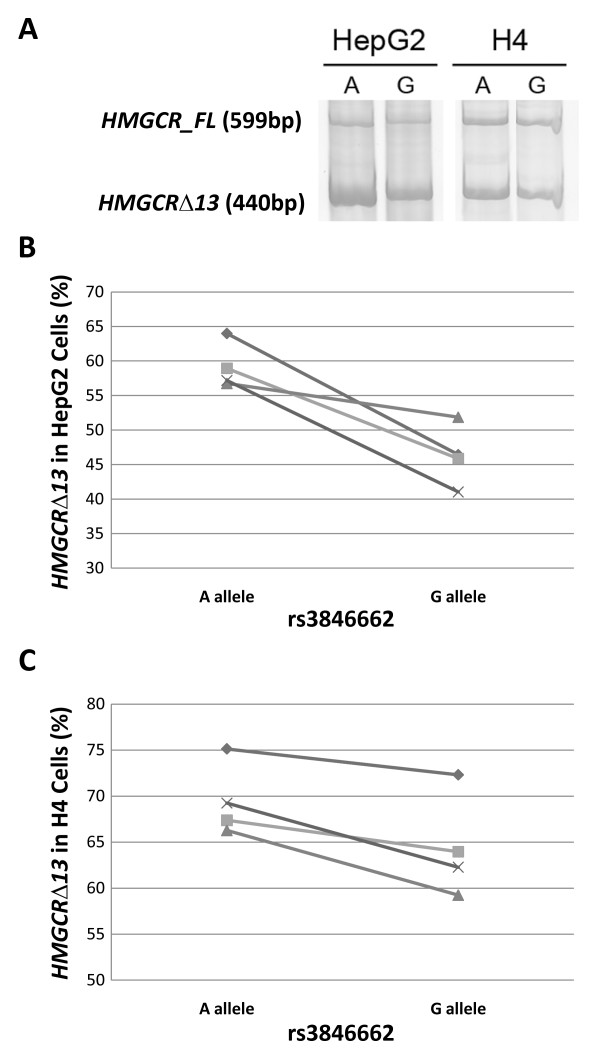
**Rs3846662 functionally modulates percent *HMGCRΔ13 in vitro *in HepG2 and H4 cells**. Cells were transfected with *HMGCR *exon 12-14 mini-genes that contained either the A or G allele of rs3846662. Vector-derived *HMGCR *isoforms were separated by PAGE and visualized by using SYGR-gold fluorescence **(A)**. The G allele of rs3846662 was consistently associated with increased retention of *HMGCR *exon 13 in both HepG2 **(B) **and H4 **(C) **cell lines relative to the A allele (p = 0.02, Student's paired t-test).

To compare *HMGCR *exon 13 splicing in homogeneous cell types reflective of liver and brain, we tested the effects of rs3846662 on *HMGCR *minigene splicing in hepatocellular carcinoma HepG2 and neuroglioma H4 cell lines. Although ectopic gene expression could cause spurious results due to over-expression per se, mini-gene transfection studies are commonly used to assess splicing-related mechanisms [32,33]. In both cell lines, the rs3846662_G allele was associated with increased retention of *HMGCR *exon 13 relative to the rs3846662_A allele (Figure 6A). In HepG2 cells, the rs3846662_G allele was associated with 12.9 ± 5.7% more *HMGCR *exon 13 retention (p = 0.02, Figure 6B). Similarly, in H4 cells, the rs3846662_G allele was associated with 5.1 ± 2.3% more *HMGCR *exon 13 retention (p = 0.02, Figure 6C). In summation, rs3846662 is associated with *HMGCR *exon 13 splicing in human liver and in cell lines derived from liver and brain.

## Discussion

The primary findings of our study are that: (1) SNPs unequivocally associated with plasma cholesterol demonstrate a significant global association with AD risk, (2) rs3846662 and rs1532085 are associated with AD age-of-onset, (3) rs3846662 is associated with *HMGCR *exon 13 splicing in human liver *in vivo *and in brain and liver-derived cell lines *in vitro*.

Our understanding of CNS cholesterol homeostasis is in its infancy relative to peripheral cholesterol homeostasis [34-37]. CNS cholesterol, under normal physiologic conditions, is maintained as a separate pool from plasma cholesterol by the blood brain barrier [38]. The SNPs studied here were chosen because they were unequivocally associated with plasma cholesterol per se [12-14,39]. Hence, these SNPs, which also are globally associated with AD risk, may influence AD via (1) peripheral effects on vasculature, and/or (2) direct effects on CNS cholesterol homeostasis.

Two lines of evidence support the possibility that these SNPs influence AD risk via peripheral or vascular effects. First, the genes associated with the majority of the tested SNPs are expressed at low levels in the brain compared to peripheral tissues, e.g., MMAB/MVK, TIMD4/HAVCR1, LIPC and CETP [40]. Hence, SNPs in these genes are unlikely to influence CNS cholesterol. Second, these SNPs are capable of impacting human disease in the periphery, as they have been associated with coronary artery disease [15,23,41]. For example, rs3846662 was associated with myocardial infarction risk in an Asian population [42]. However, SNPs which have the largest effects on plasma cholesterol did not exhibit the most significant associations with AD, as perhaps would be expected if plasma cholesterol is indeed the primary risk factor for AD [15-23].

The primary evidence supporting a role for at least a subset of the SNPs to act centrally is that *APOE *and *HMGCR *are both expressed at relatively high levels in the brain [40]. Regarding *HMGCR*, rs3846662 is associated with *HMGCR *exon 13 splicing *in vivo *in human liver and *in vitro *in brain and liver-derived cell lines, as well as lymphocytes [25]. Rs3846662 is not significantly associated with *HMGCR *exon 13 splicing in brain, although rs3846662 exhibits a similar trend as in liver. Thus, we speculate that rs3846662 may yet be functional in brain but its effects on *HMGCR *exon 13 splicing is confounded by cell-type heterogeneity and differential expression of RNA splicing factors in the CNS that our synaptophysin: GFAP model has not captured. Thus rs3846662_G may function in brain to increase *HMGCR *exon 13 retention, as observed in liver, although this effect is not discernible on the background of variation in *HMGCR *splicing in multiple brain cell types. Hence, SNPs related to *APOE *and *HMGCR *may act within the CNS. However, central and peripheral SNP effects are not necessarily mutually exclusive [43-45]. A future GWAS of CNS cholesterol would further clarify the role of SNPs in brain cholesterol metabolism and AD risk.

**Figure 7:**
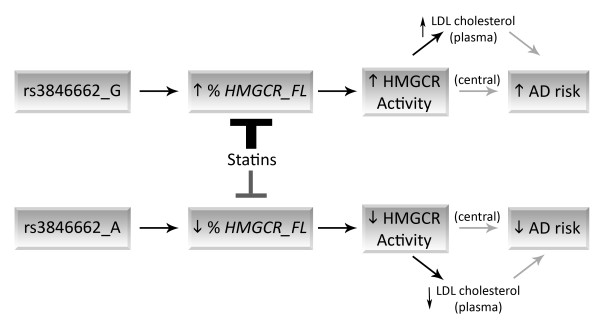
**The alleles of rs3846662 differentially affect *HMGCR *exon 13 splicing, statin responsiveness, LDL cholesterol and potentially AD risk**. Individuals carrying the rs3846662_G allele are prone to retain *HMGCR *exon 13, resulting in a greater proportion full-length *HMGCR *mRNA (% *HMGCR_FL*) and thus higher LDL cholesterol as a result of increased cellular HMGCR activity. Consequently, AD risk may be increased in these individuals due to a peripheral effect on plasma LDL and/or a central effect on HMGCR. Individuals carrying rs3846662_G, who are prone to both increased LDL and AD risk, are also predicted to be more responsive to stain therapy than individuals homozygous for the rs3846662_A allele (who tend to inefficiently splice HMGCR exon 13). Thus, rs3846662 genotype may not only increase AD risk but may also help separate potential responders from non-responders in statin trials to prevent AD.

Lastly, our finding regarding rs3846662 may contribute to understanding of the therapeutic potential of statins in AD. The rs3846662_A allele is associated with decreased HMGCR activity as reflected by decreased LDL-cholesterol (Figure 7) [15,17,18,25]. Statins also inhibit HMG-CoA reductase and robustly reduce LDL-cholesterol. Additionally, statins have been associated with reduced AD risk in multiple retrospective studies [46-48]. However, recent studies evaluating statins and AD have provided mixed results [29,30,49-52]. While the lack of consistent results could be accounted for by factors such as trial duration and variation in statin blood-brain barrier permeability, the genetics of *HMGCR *are also known to modulate statin-responsiveness, i.e., rs3846662_A is associated with a blunted response to statin therapy (Figure 7) [24,53,54]. This phenomenon has been attributed to an increased proportion of *HMGCR *lacking exon 13, which encodes a portion of both the active site of the enzyme and the statin binding site [55-57]. Hence, rs3846662_A homozygous individuals may be less likely to respond to statins in AD trials. Conversely, rs3846662_G carriers are more likely to respond to statin therapy in AD trials. Furthermore, rs3846662_G carriers appear to be at greater risk for AD, and thus have greater benefit from statin therapy. Hence, prevention trials of statin therapy for AD may benefit from consideration of rs3846662 genotype to better predict responders from non-responders.

## Conclusions

In conclusion, we report that SNPs which are robustly associated with peripheral cholesterol are globally associated with AD risk. Two SNPs in particular, rs3846662 and rs1532085, are associated with the age of AD onset. We also propose a model wherein rs3846662 acts to modulate *HMGCR *exon 13 splicing, plasma cholesterol and AD risk. Essentially, carriers of the rs3846662_G allele retain exon 13 of *HMGCR *more efficiently than rs3846662_A carriers. This relative increase in production of full-length *HMGCR *leads to increased basal cholesterol and, over the lifespan of an individual, increased AD risk. Lastly, rs3846662_G individuals appear to be at greater risk for AD and to be more likely to benefit from statin therapy to reduce AD risk.

## Methods

### SNP Selection

We performed a review of GWAS literature pertinent to human plasma cholesterol phenotypes by querying the Human Genome (HuGE) Navigator http://www.hugenavigator.net with the search term "cholesterol" and then focusing upon the traits of total cholesterol, LDL and HDL [58]. We identified 77 unique SNPs, located in or near 56 genes, associated with plasma total cholesterol, LDL and HDL. For each of these SNPs, a significant association with plasma cholesterol was observed in an initial study population and at least one confirmatory population. To limit multiple testing issues, we set a cutoff for significance of p < 1 × 10^-10^, which yielded 50 SNPs. These data were further filtered based on linkage disequilibrium. SNPs found to be in strong LD (r^2 ^> 0.8 according to the CEU HapMap population) were considered to be redundant and only the most significantly cholesterol-associated SNP was retained for further analysis [59]. As a result, our final dataset represented 18 unique SNPs residing in or near a total of 34 different genes. One of these SNPs, rs157580, is located in the gene *TOMM40 *and resides within a haplotypic block that includes the *APOE *gene. Rs157580 has been previously associated with AD and thus was considered a positive control for AD association in the present study [60,61].

### Populations and Genotyping

This work was performed in compliance with the Helsinki Declaration, with the approval of the Mayo Clinic and University of Kentucky Institutional Review Boards. Testing for an association between the eighteen cholesterol-associated SNPs and AD was performed by using a case-control population consisting of 1,940 AD and 3,925 non-AD subjects that has been previously described [62,63]. Briefly, we utilized subjects recruited for the Mayo Clinic case-control series that consisted of Caucasian individuals residing within the United States. Subjects were pooled from three separate series, including two having received clinical diagnoses of probable AD according to NINCDS-ADRDA criteria from Jacksonville, FL (JS) and Rochester, MN (RS) and age-matched controls with a score of 0 on the Clinical Dementia Rating scale. The third population was taken from the Mayo Clinic Jacksonville, FL brain bank (AUT) and had received autopsy-confirmed diagnoses of AD (NINCDS-ADRDA, Braak score > 4.0) or were chosen as controls (Braak < 2.5, not including other unrelated pathology).

DNA from these individuals was used to evaluate an association between cholesterol-associated SNPs and AD in three phases. Phase 1 was designed to identify cholesterol-associated SNPs that are nominally associated with AD. Phase 2 was designed to follow up nominally significant Phase 1 SNPs, focusing upon SNPs that had also been associated with gene regulation. Phase 3 tested for SNP association with AD in a combined population of both Phase 1 and Phase 2 subjects to maximize statistical power.

Phase 1 included subjects pooled from the JS, RS and AUT series and, after quality control screening, consisted of 843 AD and 1,264 non-AD subjects. Average ages of AD and non-AD individuals were 74.3 (SD = 4.5) and 72.4 (SD = 4.6) years, respectively. In the event that a cholesterol-associated SNP from GWAS was not available by using this platform an appropriate proxy SNP was selected using SNAPproxy, a HapMap based proxy search (http://www.broadinstitute.org/mpg/snap/; [64]). Phase 2 consisted of 1,097 AD and 2,261 non-AD subjects pooled from the JS, RS and AUT series with average ages of 80.2 (SD = 7.9) and 82.4 (SD = 5.6) years, respectively.

Phase 1 samples were genotyped by using HumanHap300-Duo Genotyping BeadChips processed with an Illumina BeadLab station (Illumina, San Diego, CA) at the Mayo Clinic Genotyping Shared Resource center (Rochester, MN). Phase 2 samples were genotyped by using SEQUENOM MassARRAY iPLEX Platform (Sequenom, San Diego, CA). All samples were subject to strict quality control including elimination of samples with call rates < 90%, MAF < 0.01, H-W p < 0.001, discrepancy between reported and genotyped sex, cryptic relatedness and discordance upon visual inspection of genotype clusters.

### AD Association Testing

Association testing for Phase 1 was performed using PLINK software to generate odds ratios (OR)s, 95% confidence intervals (CI)s and uncorrected p-values based on additive logistic regression models http://pngu.mgh.harvard.edu/purcell/plink[65]. Of the eighteen cholesterol-associated SNPs we identified in GWAS literature, rs157580 was considered to be a positive control for association with AD and thus was not considered regarding the number of tests performed. The overall association between these seventeen SNPs and AD was tested for global significance using multivariable logistic regression.

Phase 2 AD-SNP association testing was performed by using three SNPs identified in Phase 1 that were nominally associated or trended with AD (p < 0.1) and that had also been reported as having an association with gene regulation [24-27]. PLINK software was again used to generate additive logistic regression models for SNP-AD association testing.

For phase 3, we maximized statistical power by testing for association between AD and rs3846662, rs1532085 or rs7298565 in the Phase 1 and Phase 2 populations combined. Association testing was done using additive logistic regression models generated in PLINK. Rs3846662 and rs1532085 were also tested for their effect on AD age-of-onset using Cox regression models in SPSS v.18 software (IBM, Somers, NY). For each SNP, genotypes with similar effects were combined and tested against the third genotype for association with AD age-of onset using SPSS.

With a total of ~2,000 AD cases and ~4,000 non-AD controls, we have greater than 80% power to detect an association between AD and rs3846662 or rs1532085. Power calculations were performed by using the method developed by *Purcell S et al*. http://pngu.mgh.harvard.edu/~purcell/gpc/cc2.html[66].

### Human Tissue

Human brain tissue from the anterior cingulate was generously provided by the Sanders-Brown Alzheimer's Disease Center Neuropathology Core and have been described elsewhere [67]. The samples were from deceased individuals with an average age at death of 82.4 ± 8.7 (mean ± SD) years for non-AD and 81.7 ± 6.2 years for AD subjects. The average postmortem interval (PMI) for non-AD and AD subjects was 2.8 ± 0.8 and 3.4 ± 0.6 hours, respectively. Non-AD and AD samples were comprised of 48% and 55% female subjects. MMSE scores were, on average, 28.4 ± 1.6 for non-AD subjects and 11.9 ± 8.0 for AD subjects.

Human liver samples were obtained from the Brain and Tissue Bank for Developmental Disorders (Baltimore, MD) and have been previously described [68]. The samples were from deceased individuals with an average overall age at death of 26.9 ± 9.0 years of age. The average age at death was similar for women (27.3 ± 8.5 years, n = 17) and for men (26.6 ± 9.4 years, n = 23). The average PMI for women was 12.9 ± 4.5 hours while the average PMI for men was 10.0 ± 3.0 hours.

### Evaluation of rs3846662 effect on HMGCR exon 13 splicing efficiency in vivo

To evaluate the association between rs3846662 and *HMGCR *exon 13 splicing in human brain and liver tissue, genomic DNA and RNA were prepared from human tissue samples; cDNA was then reverse transcribed as reported previously [68,69]. Isoform specific primers were designed to amplify either *HMGCR *that contains exon 13 (*HMGCR_FL*) or lacks exon 13 *(HMGCRΔ13)*. To accomplish this we designed a forward primer specific to *HMGCR *exon 12 (5'-TGAGCGTGGTGTATCTATTCG-3') and reverse primers specific to either *HMGCR *exon 13 (5'-GGCCACAAGACAACCTTCTG-3') or the junction of *HMGCR *exons 12 and 14 (5'-CCTCCACCAAGCAAGGAGTA-3') to amplify *HMGCR_FL *and *HMGCRΔ13*, respectively.

Quantitative real-time PCR was performed on an MJ Opticon 4 thermal cycler (Biorad, Hercules, CA) by using 20 ng of subject cDNA together with 10 μl of SYBR green reagent (Quanta Biosciences, Gaithersburg, MD), 10 μl of H_2_O and forward and reverse primers, each at 1 μM final concentration. Cycling conditions consisted of a 3 minute denaturation step at 95°C followed by 40 cycles of denaturation for 15 seconds at 95°C and annealing/extension for 45 seconds at 60°C. A final melting curve was used to assess amplification fidelity in conjunction with subsequent inspection of PCR products on 8% TBE-PAGE gel (Sigma) stained with SYBR gold (Invitrogen, Carlsbad, CA).

Isoform-specific PCR products were generated, quantified at A260 and used to generate standard curves consisting of 10-fold serial dilutions that were amplified alongside samples with each PCR run. These standard curves allowed for monitoring reaction efficiency and absolute quantification of *HMGCR *isoforms. *HMGCR *expression was normalized to the geometric mean of internal reference genes, *HPRT *and *RPL32*, for each individual sample as we have described previously [70-72] and compared to the expression of synaptophysin and GFAP, which were quantified as described previously [73].

Testing for an association between rs3846662 and HMGCR expression and splicing was performed by using linear regression. Furthermore, the covariates age, sex and PMI were included in the analysis to correct for confounding differences in these demographics between brain and liver samples. All tests were performed using SPSS v.18 software.

### Immunofluorescent staining of HMGCR

Paraffin-embedded blocks of human anterior cingulate were sliced at a thickness of 5 μm. Sections were fixed to glass slides prior to deparaffinization in a series of xylene/EtOH/H_2_O. Deparaffinized sections then submersed in pH 6 citrate buffer containing emulsifiers (Cell Marque, Cat # CMX633) and subject to antigen retrieval in a decloaking chamber (Biocare Medical, Cat # DC2002) for 30 min at 80C then by 10 min at 75C. Following antigen retrieval, slides were rinsed with H_2_O and then 1 × TBS (pH 7.4) before blocking with 5% goat serum in 1 × TBS for one hour. Slides were then rinsed again with 1 × TBS prior to incubation with rabbit α-HMGCR (1:25 dilution, Millipore Cat. # 07-457, Lot # 28743) and either mouse α-MAP2 (1:100 dilution, Sigma) or mouse α-GFAP (1:25 dilution, ICN Biomedicals, Cat # 691102) in a solution of 2% goat serum (Sigma, Cat #G6767) overnight at 4°C. Slides were rinsed with 1 × TBS and then treated with Alexa Fluor 488 goat α-mouse (Mol. Probes, Cat # 11029) and Alexa Fluor 568 goat α-rabbit (Mol. Probes, Cat # 11036) secondary antibodies (1:200 dilution) in 0.15% goat serum and 1 × TBS for one hour at room temperature. Labeled slides were then washed in 1 × TBS before treatment with Autofluorescence Eliminator Reagent (Chemicon, Cat # 2160) per the manufacturer's instructions. Slides were mounted using an anti-fading solution containing DAPI (Vectashield, Cat # H-1200), coverslipped, and visualized on a Nikon Diaphot fluorescence microscope.

### In vitro minigene studies

*HMGCR *exon 12-14 mini-gene vectors harboring either the A or G allele of rs3846662 were a kind gift of Dr. Jan L. Breslow (The Rockefeller University) and have been previously described [25]. The sequence of the *HMGCR *inserts differs only at rs3846662 (A/G).

To evaluate the effect of rs3846662 in homogenous cell types, as compared to the heterogeneous cellular environment of human brain and liver, we chose brain and liver-derived cell lines for transfection with *HMGCR *mini-genes. H4 human neuroglioma cells were maintained in Opti-MEM (Invitrogen) supplemented with a final concentration of 10% fetal bovine serum (FBS), 50 U/ml penicillin and 50 μg/ml streptomycin (P/S). HepG2 human hepatocellular carcinoma cells were grown in DMEM (Invitrogen) supplemented with a final concentration of 10% FBS and 1% P/S. Both cell lines were incubated at 37°C in a humidified 5% CO_2 _environment.

Both H4 and HepG2 cells were seeded in 6-well plates (2 × 10^5 ^cells/well) containing either antibiotic-free Opti-MEM or DMEM media and 10% FBS, respectively. Cells were allowed to grow for 24 hours prior to transfection with 1 μg of either allele-specific *HMGCR *mini-gene vector in 6 μl of FuGENE6 reagent (Roche Applied Sciences, Switzerland) and 94 μl of Opti-MEM (for H4 cells) or DMEM (for HepG2 cells), per the manufacturer's recommendations. Total RNA was prepared from cells twenty-four hours after transfection (RNeasy Mini Kit, QIAGEN) and quantified at A260 before being reverse transcribed using random hexamers per the manufacture's directions (SuperScript III, Invitrogen). Transfections were performed in triplicate for each allele.

Conventional PCR amplification of cDNA transcripts from *HMGCR *mini-genes was used to assess the splicing efficiency of *HMGCR *exon 13 *in vitro *as a function of rs3846662. A reverse primer specific to *HMGCR *exon 14 (5'-AGTGCTGTCAAATGCCTCCT-3') and a forward primer specific to the expressed, upstream portion of the pSPL3 exon-trapping vector (5'-TCTGAGTCACCTGGACAACC-3') were used to amplify vector-derived *HMGCR_FL *and *HMGCRΔ13*. Conventional PCR and gel-based methods were used to quantify vector-derived *HMGCR *isoforms because the large PCR product size (440 bp for *HMGCRΔ13 *and 599 bp for *HMGCR_FL*) precluded real time PCR analysis. Amplification was performed for 32 cycles following an initial denaturation at 95°C for 5 minutes followed by cycling of 95°C denaturation for 30 seconds, 60°C annealing for 30 seconds and 72°C extension for 1 minute, with a final extension at 72°C for 2 minutes. PCR products were resolved using 8% TBE-PAGE gel electrophoresis and visualized following staining with SYBR-gold and fluorescent image capture (Fuji FLA2000). Vector-derived *HMGCR_FL *and *HMGCRΔ13 *were identified by size and confirmed by sequencing (Davis Sequencing, Davis, CA). The amounts of *HMGCR_FL *and *HMGCRΔ13 *were quantified by fluorescent densitometry, corrected for background and normalized for size as described previously [70]. The ratios of *HMGCR_FL *and *HMGCRΔ13 *for each transfection were then calculated to assess splicing efficiency and analyzed for their association with rs3846662 by using a paired Student's t-test for each transfection experiment.

## List of abbreviations

AD: Alzheimer's disease; GWAS: genome wide association study; SNP: single nucleotide polymorphism; HMGCR: HMG-CoA reductase

## Competing interests

The authors declare that they have no competing interests.

## Authors' contributions

CRS and SE conceived all experiments and wrote the manuscript. CRS performed RT-PCR of *HMGCR*, rs3846662 genotyping of human brain and liver, transfection of mini-genes and immunofluorescence study. CRS and SE performed statistical analyses of human bran and liver data. FZ performed all genotyping of phase 2 case-control samples. SGY, FZ and CRS performed statistical analysis of the association between cholesterol-associated SNPs and AD GWAS data. All authors have read and approve of the final manuscript.
